# Suxamethonium induces a prompt increase in the bispectral index

**DOI:** 10.1097/MD.0000000000006670

**Published:** 2017-04-21

**Authors:** Jong Bum Choi, Se Hee Na, Sook Young Lee, Jong Yeop Kim, Sung Yong Park, Ji Eun Kim, Seungbae Hong, Jiwon An, Chung Hoon Park, Yong Chan Kim, Woo Young Park

**Affiliations:** a Department of Anesthesiology and Pain Medicine, Ajou University, College of Medicine, Suwon; b Department of Anesthesiology and Pain Medicine, Gangnam Severance Hospital, Yonsei University College of Medicine, Seoul; c Department of Anesthesiology and Pain Medicine, Gwangmyeong Saeum Hospital, Gwangmyeong, Korea; d Department of Anesthesia, Sheikh Khalifa Specialty Hospital, Ras Al Khaimah, United Arab Emirates; e Department of Anesthesiology and Pain Medicine, Seoul National University College of Medicine, Seoul, Korea.

**Keywords:** anesthesia, bispectral index, suxamethonium

## Abstract

Upon inducting general anesthesia in the operating room, we have observed a prompt increase in the bispectral index (BIS) after the intravenous injection of suxamethonium. We hypothesized that the cause of this BIS increase is muscle hyperactivity owing to fasciculation. However, no reports have been published regarding this abrupt increase in the BIS upon the induction of general anesthesia by suxamethonium. To investigate the degree of change in the BIS in patients receiving anesthesia with suxamethonium, we performed a prospective observational study of 63 participants who underwent closed reduction for nasal bone fracture. Anesthesia was induced by the total intravenous administration of anesthetics and 1.5 mg kg^−1^ of suxamethonium was injected intravenously upon achieving BIS between 45 and 55. Intubation was performed after fasciculation. Electromyograms and BIS values were recorded from the induction of suxamethonium until 15 minutes after intubation. The mean BIS values were 95.4, 48.5, and 69.3 before induction, before the intravenous injection of suxamethonium, and immediately after fasciculation, respectively. The BIS value immediately after fasciculation (69.3 ± 10.6) was significantly higher than the cutoff BIS value of 60 (*P* < .001). Although fasciculation after the intravenous injection of suxamethonium resulted in the prompt increase of the BIS to values over 60, none of the participants was awake during surgery. In conclusion, the administration of suxamethonium resulted in the postfasciculation increase of the BIS to an average value of 69.3 without affecting the patient's state of consciousness.

## 1 Introduction

The bispectral index (BIS) is commonly used to measure the sedation level and effects of anesthesia.^[1]^ The BIS score, which ranges from 0 to 100, shows changes in the hypnotic level based on electroencephalogram (EEG) readings.^[2]^ However, the BIS is known to be influenced by external factors such as electromyography (EMG) and warming machines.^[3,4]^ In addition, nondepolarizing neuromuscular blocking agents have been shown to decrease the BIS.^[5]^

Suxamethonium is a unique depolarizing neuromuscular blocking agent with clinical applications. Messner et al^[6]^ reported that the intravenous injection of suxamethonium in 3 awake volunteers decreased the BIS. In contrast, in our clinical practice, we observed that upon the induction of general anesthesia with suxamethonium, the BIS increased abruptly after fasciculation and then decreased. This abrupt increase in the BIS might be related to fasciculation resulting from the increase in muscle activity. However, this phenomenon of an abrupt increase in the BIS upon general anesthesia induction has not been reported previously in the literature. According to our experience, the time at which the BIS increases corresponds to the time of intubation. The abrupt BIS increase might make anesthesiologists hesitate to perform intubation or cause them to increase the dose of anesthetic agents out of fear that their patient may regain consciousness. Therefore, in the present study, we investigated the extent of the BIS change upon inducing anesthesia with suxamethonium.

## 2 Methods

### 2.1 Ethical approval

Ethical approval for this study was obtained from the institutional review board of Gangnam Severance Hospital in Seoul, Republic of Korea (3-2012-0033). Patients provided written informed consent for their data to be analyzed and published for research purposes.

### 2.2 Inclusion and exclusion criteria

The inclusion criteria for this study were as follows: operation time with general anesthesia <15 minutes; American Society of Anesthesiologists (ASA) physical status I or II; age between 20 and 65 years; and literacy. The exclusion criteria were as follows: operation time with general anesthesia ≥15 minutes; massive bleeding; inability to attach a BIS monitor; myopathy; contraindication to suxamethonium; ASA physical status III, IV, V, or VI; and illiteracy.

### 2.3 Experimental timeline

This study included 63 patients who underwent closed reduction for nasal bone fracture under general anesthesia between July 2012 and January 2013. None of the patients was premedicated. In the operating room, after standard monitoring including electrocardiography, pulse oximetry, and noninvasive blood pressure measurement, a BIS-monitoring electrode (Bis quatro, Aspect Medical Systems, Norwood, MA) was placed on the forehead of the patient after careful cleaning of the skin according to the manufacturer's instructions. The electrode was then attached to a BIS monitor (Model A-3000 vista, Aspect Medical Systems). The BIS monitor is a quantitative EEG device that uses a proprietary algorithm to analyze the electrical signal derived from a frontal electrode array. This analysis generates a number between 0 and 100. Values >80 indicate that the patient is awake, while values between 60 and 80 indicate sedation to the extent that the patient may respond purposefully to a stimulus. Values between 40 and 60 are thought to reflect a level of unconsciousness appropriate for surgery.^[7]^ Therefore, we used a cutoff value of 60, because patients could still respond to a stimulus at BIS values >60. Anesthesia was induced with 2% propofol and remifentanil by total intravenous administration, after which mask ventilation was applied manually. In the case of mask ventilation difficulties, the oral airway was used for the same purpose. Mask ventilation was continued until the BIS values were maintained between 45 and 55 over 2 minutes; then, 1.5 mg kg^−1^ suxamethonium was injected intravenously. After fasciculation, intubation was performed once the patient was paralyzed. The BIS and EMG readings were acquired immediately before the induction of anesthesia, before the injection of suxamethonium, immediately after fasciculation, and at 1, 2, 3, 4, 5, 10, and 15 minutes after fasciculation. The primary endpoint was an increase in the maximal BIS to values >60 immediately after fasciculation. The secondary endpoint was intraoperative arousal, which was confirmed by postoperatively asking patients about the arousal levels they experienced during surgery. The baseline BIS and EMG values, which were acquired immediately before the injection of suxamethonium, were compared with those acquired at each time point.

### 2.4 Statistical analysis

Data obtained from a previous pilot study at our hospital indicated that 63 patients would be required to achieve α and β-errors of 0.05 and 0.2, respectively, assuming differences in the BIS of 64.2 (mean BIS of the pilot study) and 60, respectively. Statistical analysis was performed using the SAS v. 9.2 software (SAS Institute Inc., Cary, NC). All data are presented as the mean (standard deviation [SD]). To compare the maximal BIS values immediately after fasciculation with the baseline values, we used the one-sample *t*-test. Differences in the BIS and EMG values between baseline and the other examined time points were analyzed with a linear mixed-model analysis. Probability values < .05 were considered statistically significant.

## 3 Results

**Figure 1:**
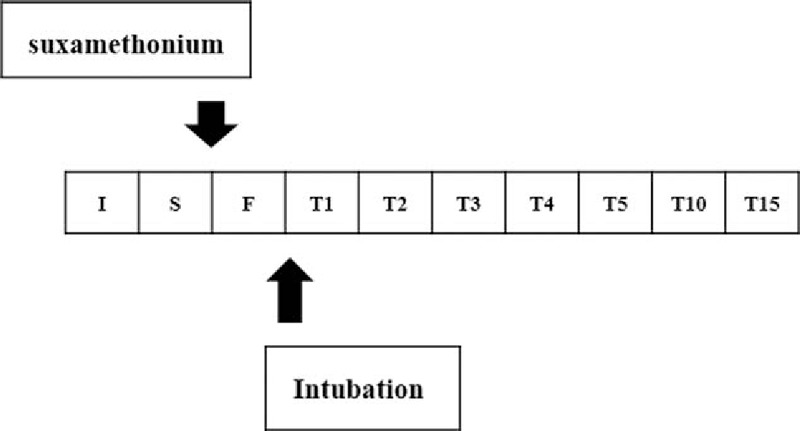
Experimental time points. I: before the injection of hypnotics; S: immediately before the injection of suxamethonium; F: immediately after fasciculation; T1–5: 1–5 minutes after fasciculation; T10: 10 minutes after fasciculation; T15: 15 minutes after fasciculation.

**Figure 2:**
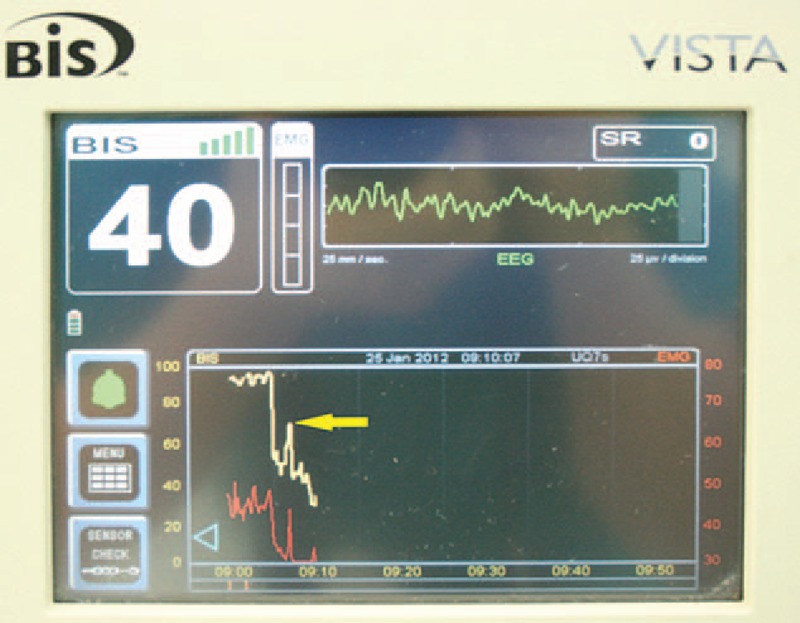
Representative BIS readings in a single patient. Arrow = abrupt increase in the BIS after fasciculation, BIS = bispectral index, EMG = electromyography, red line = EMG values.

**Figure 3:**
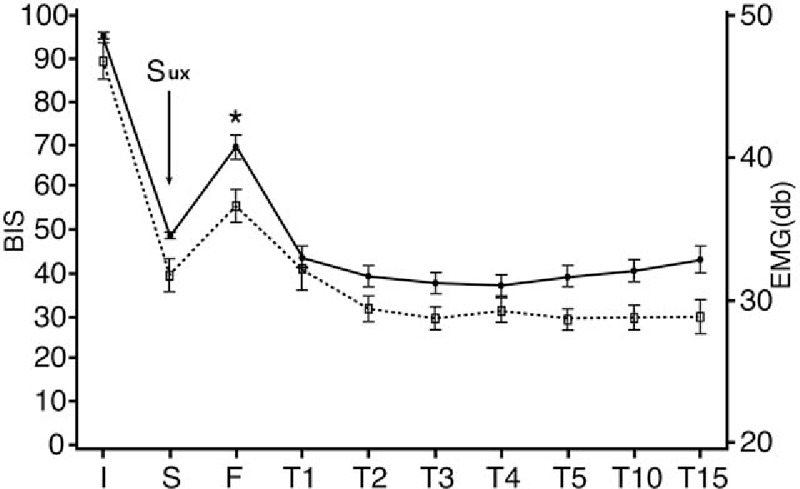
Changes in the BIS (solid lines) and EMG (dashed lines) values after the injection of suxamethonium. Data on the *y*-axes indicate the BIS and EMG values at each time point from the time of suxamethonium injection. Error bars indicate standard deviation. ∗BIS value statistically significantly higher than 60 (*P* < .05). BIS = bispectral index, downward arrow = the time of intravenous suxamethonium injection, EMG = electromyography, Sux = suxamethonium, I: before the injection of hypnotics; S: immediately before the injection of suxamethonium; F: immediately after fasciculation; T1–5: 1–5 minutes after fasciculation; T10: 10 minutes after fasciculation; T15: 15 minutes after fasciculation.

Patient characteristics are presented in Table 1. The flow chart of the protocol is presented in Fig. 1. The mean (SD) maximal BIS value immediately after fasciculation was 69.3 (10.6), which was statistically significantly higher than the cutoff value of 60 (*P* < .001; Figs. 2 and 3). The maximum BIS values immediately after fasciculation were 80 to 89 in 9 patients, 70 to 79 in 28, 60 to 69 in 14, and <60 in 12. None of the patients was awake during surgery. The baseline BIS values were significantly different from those measured immediately and at 1, 2, 3, 4, 5, 10, and 15 minutes after fasciculation (Table 2). The differences in the EMG values between baseline and each of the examined time points, with the exception of 1 minutes after fasciculation, were significant (Table 3).

**Table 1:**
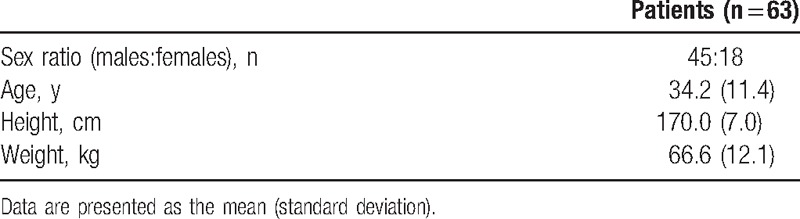
Patient characteristics.

**Table 2:**
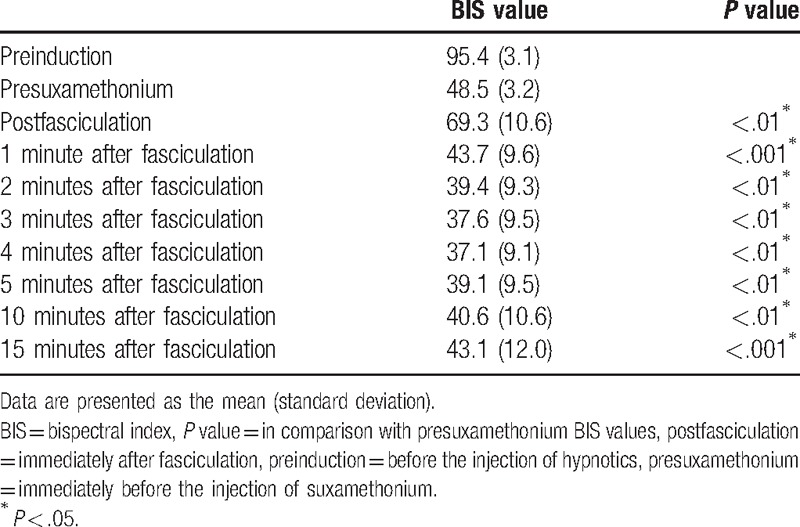
Changes in BIS values.

**Table 3:**
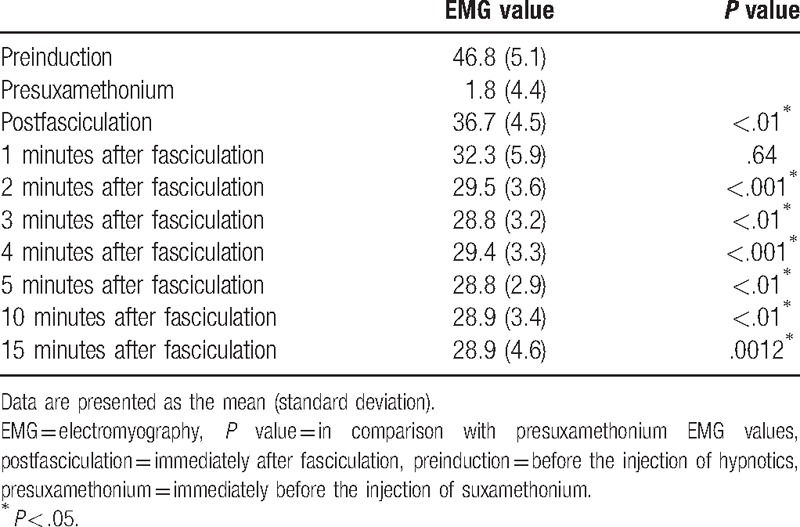
Changes in EMG values.

## 4 Discussion

In this study, we demonstrated that inducing anesthesia with suxamethonium, a depolarizing neuromuscular blocker, abruptly increased the BIS immediately after fasciculation.

Currently, the abovementioned report by Messner et al^[6]^ is the definitive study regarding BIS variations induced by suxamethonium. In that study, the authors found that injecting individuals with suxamethonium while awake induced a decrease in the BIS in all 3 cases.^[6]^ However, because these volunteers were not anesthetized, the BIS values before suxamethonium injection were >90, which might explain why the authors did not observe an abrupt increase in the BIS immediately after fasciculation. On the other hand, no other studies on the variations in the BIS upon suxamethonium administration have demonstrated increases in the BIS.^[8–10]^ For instance, in their study examining variations in the BIS in 10 conscious patients, Schuller et al^[11]^ reported that the administration of neuromuscular blocking agents decreased the BIS values; however, the authors did not comment on whether there was an increase in the BIS after fasciculation.

Suxamethonium is a depolarizing neuromuscular blocker known to induce fasciculation and increase muscle activity.^[12]^ In the present study, we demonstrated that suxamethonium administration resulted in an abrupt increase in the BIS immediately after fasciculation. The rate of increase was rapid, and its duration was short. Suxamethonium is often used in patients requiring rapid sequence intubation or short, simple surgery. In our experience, the induction of anesthesia with suxamethonium in pregnant women before a Caesarean section results in an abrupt increase in the BIS. Consequently, we were unable to perform rapid sequence intubation in these cases; instead, the patients were ventilated manually with a mask, and intubation was performed after the BIS decreased. However, this phenomenon of a significant increase in the BIS immediately after fasciculation has not been reported previously in the literature. Here, we found that the maximal BIS (mean value, 69.3) was >60 in 51 patients (81.0%), >70 in 37 (58.7%), and >80 in 9 (14.3%) patients.

Anesthesiologists hesitate to perform intubation in cases where the BIS values exceed 60. However, it is not clear whether the increase in the BIS immediately after fasciculation indicates a change in the hypnotic level or a change in the EMG activity. Although a previous study demonstrated that muscle relaxation does not induce changes in the level of hypnosis,^[1]^ no studies have been published on the effects of increased muscle activity on the level of hypnosis. However, based on the findings of another previous study,^[3]^ which demonstrated an increase in the BIS owing to EMG activity, we believe that the increase in BIS that we observed in the present study was induced by an increase in the EMG activity rather than by a change in the level of hypnosis. This hypothesis is supported by our findings demonstrating increases in the EMG activity after suxamethonium injection (Figs. 2 and 3).

Because this was not a comparative, randomized, double-blind study, our findings have several limitations. First, we did not include a control group in which a nondepolarizing neuromuscular block was applied. Second, we did not vary the anesthetic agent; hence, it is unclear whether the results would change under various anesthetic agents. Furthermore, our results might vary in patients who are premedicated or in patients where anesthesia is induced with inhalation agents. Third, the BIS electrod array, the kind of BIS monitor may influence the value of BIS. We did not consider. Fourth, in literatures cited about relationship suxamethonium and BIS, hypnotics were not used but in our research, we used hypnotics. We did not exclude the influences by hypnotics. Fifth, we investigated from awake state before induction to end of surgery. But we did not investigate the time of return of muscle activity from paralysis by suxamethonium. So we did not find whole relationship between BIS and EMG. Further research is required to validate our findings.

In conclusion, the administration of suxamethonium increased the average maximal BIS from 48.5 to 69.3 after fasciculation. However, this increase did not affect the patients’ state of consciousness during surgery.

## Acknowledgments

The author thanks Kyunghwa Han for the statistical analysis.
